# Polymer-Based MEMS Electromagnetic Actuator for Biomedical Application: A Review

**DOI:** 10.3390/polym12051184

**Published:** 2020-05-22

**Authors:** Jumril Yunas, Budi Mulyanti, Ida Hamidah, Muzalifah Mohd Said, Roer Eka Pawinanto, Wan Amar Fikri Wan Ali, Ayub Subandi, Azrul Azlan Hamzah, Rhonira Latif, Burhanuddin Yeop Majlis

**Affiliations:** 1 Institute of Microengineering and Nanoelectronics, Universiti Kebangsaan Malaysia, Bangi 43600, Selangor, Malaysia; p86814@siswa.ukm.edu.my (W.A.F.W.A.); p93203@siswa.ukm.edu.my (A.S.); azlanhamzah@ukm.edu.my (A.A.H.); rhonira@ukm.edu.my (R.L.); burhan@ukm.edu.my (B.Y.M.); 2 Faculty of Engineering and Vocational Education, Universitas Pendidikan Indonesia, Jl. Dr. Setiabudhi 207, Bandung 40154, Indonesia; bmulyanti@upi.edu (B.M.); idahamidah@upi.edu (I.H.); 3 Faculty of Electronics and Computer Engineering (FKEKK), Universiti Teknikal Malaysia Melaka (UTeM), Hang Tuah Jaya, Durian Tunggal 76100, Melaka, Malaysia; muzalifah@utem.edu.my; 4 Malaysia-Japan International Institute of Technology (MJIIT), Universiti Teknologi Malaysia (UTM), Kuala Lumpur 54100, Malaysia; roer.eka@gmail.com

**Keywords:** polymer composites, microelectromechanical system (MEMS), electromagnetic (EM) actuator, magnetic membrane, microfluidic, biomedical

## Abstract

In this study, we present a comprehensive review of polymer-based microelectromechanical systems (MEMS) electromagnetic (EM) actuators and their implementation in the biomedical engineering field. The purpose of this review is to provide a comprehensive summary on the latest development of electromagnetically driven microactuators for biomedical application that is focused on the movable structure development made of polymers. The discussion does not only focus on the polymeric material part itself, but also covers the basic mechanism of the mechanical actuation, the state of the art of the membrane development and its application. In this review, a clear description about the scheme used to drive the micro-actuators, the concept of mechanical deformation of the movable magnetic membrane and its interaction with actuator system are described in detail. Some comparisons are made to scrutinize the advantages and disadvantages of electromagnetic MEMS actuator performance. The previous studies and explanations on the technology used to fabricate the polymer-based membrane component of the electromagnetically driven microactuators system are presented. The study on the materials and the synthesis method implemented during the fabrication process for the development of the actuators are also briefly described in this review. Furthermore, potential applications of polymer-based MEMS EM actuators in the biomedical field are also described. It is concluded that much progress has been made in the material development of the actuator. The technology trend has moved from the use of bulk magnetic material to using magnetic polymer composites. The future benefits of these compact flexible material employments will offer a wide range of potential implementation of polymer composites in wearable and portable biomedical device applications.

## 1. Introduction

Over the past few years, there has been an increasing demand on the employment of flexible materials for various applications in biomedical field. This has led to the significant growth of the movable structure development [1,2]. The flexible material having good mechanical properties with high surface strength and high elasticity has enabled tremendous innovation in the development of microelectromechanical systems (MEMS) devices in which the electrical and mechanical property of the material are the most important characteristics of the technology [3]. One of the most interesting materials is polymer that currently can be found in various biomedical instrumentation due to its excellent mechanical properties, compactness, precise control and biocompatibility as well [4].

The flexibility characteristic of polymer is beneficial in obtaining large and controlled structure deformation of the movable parts. These movable parts include diaphragm (thin membrane), pillars, cantilevers or the combination of pillars and movable structures [5,6]. This class of functional material plays very important role in the development of MEMS electromagnetic (EM) actuators, for example the microfluidic delivery system found in drug delivery, bio-cell preparation system and lab on chip [7]. The system can also include micropumps, microvalve, micromixer, microgripper and micromanipulators [8,9,10,11].

Studies on electromagnetically driven MEMS actuators in the field of biomedical instrumentation are currently increasingly popular in which the improvements of the mechanical structures and the material properties of the movable part became the most interesting topics. The development studies were done in order to enable efficient and precise structure movement for control, manipulation or analysis purpose of the biomedical samples [12,13]. These studies also have led to the invention of flexible structure possessing sensitive interaction with magnetic induction, to be the most important mechanism in electromagnetic actuation. The moving structures should be made of soft and elastic material, able to continuously vibrate and capable of reacting to mechanical pressure and magnetic field exposures [14].

Several reports have been recently published to introduce the interaction between magnetic flux generated from electromagnetic coil and rotating magnet field [15,16]. This interaction is the basic principal operation of the electromagnetic actuator that produces magnetic force to enable the movement of a movable structure. The basic electromagnetic actuator structure consists of a flexible movable membrane, electromagnetic coil, magnetic chamber or spacer and bulk permanent magnet. Initially, a thin membrane attached with permanent magnet has been the common structure used as the moving membrane of the MEMS electromagnetic actuator [17]. Unfortunately, the structure with attached bulk magnet suffers from high volume and low reliability, especially when the membrane operates in long vibration mode [18]. Therefore, some innovations in the material structure have been developed in order to obtain a compact and reliable actuator.

The MEMS structures are usually made of glass, silicon, silicon nitride and metals [19,20]. Those materials are the common materials in MEMS technology due to the excellent mechanical properties and matured technology process [21]. However, silicon and glass are easy to break as they have low fracture strain which is about 0.1% [22,23,24]. Meanwhile, metals are very sensitive to chemical and environmental effect [25]. Some other disadvantages of those conventional MEMS materials, especially for the use as movable structure, are fragile and low flexibility. These drawbacks make them less favorable compared to polymers.

On the other hand, polymers in MEMS have been used since several years ago as a photosensitive material [26], sacrificial layer [27], passive structure for microchannel [28], microchamber and passive micromixer [29] and as the functional layer of micro-structured devices, such as actuators [30] and sensors [31,32]. Polymer has good mechanical properties with Young’s modulus lower than silicon and metal, which makes it highly elastic and at the same time possesses high strength [33,34,35,36,37,38]. In conjunction with MEMS actuators, the mentioned mechanical properties are useful in obtaining large membrane deformation under external magnetic stimulus. Furthermore, the most important fact is that the polymeric structure of MEMS device can be fabricated in inexpensive way, cheaper than silicon-based micro-processing cost [39,40,41].

It was also reported that microstructures working under extreme vibration condition like actuators need enhancement in terms of material quality, design and technological concepts in order to increase the lifetime and effectiveness of the structures [38]. Therefore, some magnetic polymers become more preferable as the structures will have high elasticity, easy to fabricate and photo-patternable.

Some popular polymers have been identified and explored to become the flexible material for actuation purposes. The common actuator materials that have been reported in the literatures include PMMA, parylene, polyimide and PDMS elastomer. The properties of those materials are summarized in Table 1.

## 2. MEMS Actuators

In general, MEMS actuators can be driven either by mechanical actuation or non-mechanical actuation. Mechanical actuation mechanism with a diaphragm (membrane) as the moving part is primarily utilized in MEMS devices [48]. Compared to the non-mechanical actuator, the mechanical actuator has many advantages in terms of controllability, high vibration rate and large membrane deformation [49].

A large number of mechanical microactuator devices has been demonstrated including microrelays, microvalves, optical switches and mirrors, micropumps and many others that can be found in various applications. These actuators use different mechanical actuation principle such as piezoelectric, electrostatic, electromagnetic, thermo-mechanic, thermo-pneumatic and shape memory. Table 2 shows a comprehensive analysis of MEMS mechanical actuators, describing different types of energy exchange mechanism used to obtain kinetic movement, the devices’ structure, the advantages and the typical applications of each MEMS mechanical actuator.

The major advantages of electromagnetic actuation are the generated high magnetic force that enables large membrane deflection and high tunable frequency capability. In addition, rapid generation of electromagnetic field enables membrane deformation in 2 directions with very fast vibration rate [10]. Additionally, an electromagnetic actuator is capable of precisely tuning the input power. The power consumption in EM actuators between 13 mW to 7 W is the widest range among the other types of actuators [59]. However, high power dissipation and large area consumption could be the drawbacks of the system.

Not many designs for magnetic microactuators specifically used in biomedical field are reported in literature. Table 3 shows the developed magnetic microactuator devices for biomedical application. The application of these actuators are classified into biosample delivery/transport, biosample preparation and biocell manipulation. Mainly, the actuator functioned as a microfluidic handling system for samples delivery in a drug delivery system and lab on chip. There is a high interest from industry in the implementation of the electromagnetically driven microactuators for a broad range of biomedical applications.

### Electromagnetic Actuators Principle

The basic mechanism of electromagnetic actuation involves the interaction between magnet and electromagnetic field that intensively generates the magnetic force. This interaction produces high frequency vibration of the movable structures, such as membrane and pillars, hence enables various implementation of biomedical instrumentation.

Thielicke et al., [70] explained that the actuation principle depends on structural dimension, response time, torque, max power consumption, the technology used and the applied forces. The forces are classified into 2 main groups, namely external and internal forces. Electromagnetic actuators fall in external forces category as the forces are produced from the magnetic fields interaction occurred in the gap between the stationary and moving parts.

**Figure 1:**
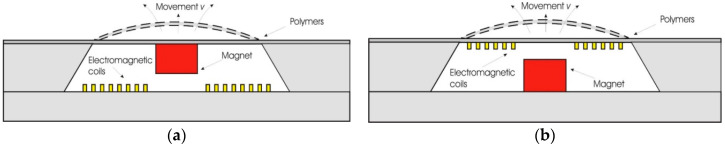
Cross sectional view of an initial electromagnetic (EM) actuator, (**a**) with magnetic membrane-based moving parts [50], (**b**) with embedded planar coil-based moving parts [74].

In general, the magnetic membrane actuation is achieved by the deformation of the movable membrane due to the generated magnetic force acting onto the membrane. The common structure of a magnetic actuator is schematically displayed in Figure 1a. The system consists of the magnetic field generator part (electromagnetic coil) and the magnetic membrane part (flexible membrane plus an attached magnet) [71,72].

Through the interaction between magnet and electromagnetic coil, a vertical magnetic force acting on the magnetic membrane with vertical magnetization on *z*-axis is generated. The magnetic force known as Lorentz force $F_{mag}$ is given by the following integral over the volume *V* of the body [73]:

(1) $$F_{mag}=M_{z}\int_{v}^{}\frac{\partial H_{z}}{\partial z}dV$$

where, *Fmag* is the magnetic force acting on the magnetic membrane, $M_{z}$ is the magnetic induction from the permanent magnet, $\frac{\partial H_{z}}{\partial z}$ is the magnetic field gradient generated by the electromagnetic coils and *dV* is the volume of the permanent magnet. The correlation between magnetic force applied on to the membrane and the resulting membrane deformation $h_{z}$ can be derived from the Equation (1):

(2) $$h_{z}=C\frac{Fmag\;l_{m}}{D}$$

where, $l_{m}$ is the membrane size, *C* is a constant depending on the shape and geometry and *D* is the material characteristics of the membrane that is defined by:

(3) $$D=\frac{E\;t_{m}{}^{2}}{12\;\left(1-v^{2}\right)}$$

where *E* is the Young’s Modulus, *v* is the Poisson’s ratio while, *t_m_* is the thickness of the membrane.

Another approach to introduce the principal of the actuation mechanism has been described by Pawinanto et al. [71] and Sugandi et al. [74]. Here, planar electromagnetic coil wires are embedded inside or attached on the movable membrane surface, as shown in Figure 1b. When an electrical current is supplied to the planar coil wires, a magnetic flux induction from the permanent magnet onto the wires is achieved. Through this induction, the magnetic force *Fmag* is generated and acting onto the membrane that finally causes the periodical actuation of the membrane structure.

At the location of the coil, magnetic field makes an angle θ with the normal surface (vertical axis) and the magnetic force (*Fmag*) between a current carrying wire and a permanent magnet can be expressed as given by [22]:

(4) $$\vec{F_{mag}}=\overset{N}{\underset{i=1}{\sum }}2\pi R_{i}I\times \vec{B_{r}}\left(R_{i}\right)\times \sin\theta$$

where *I* is the coil current, *R_i_* the radius of each turn coil, $\vec{B_{r}}$ the radial component of magnetic field in the coil plane and *θ* is angle direction of magnetic field to vertical axis. Therefore, total force for a single turn coil is given by:

(5) $$\vec{F_{mag}}=I\;(l\times \vec{B_{r}}\times \sin\theta )$$

with *l* represents the total length of a single turn coil with a radius *r*. Using both equations, we can see where the force vector direction acted. The induced electromagnetic force is principally based on the magnetic interaction between the current carrying coils, permanent magnets and flexible membrane materials [71]. It works vice versa, either the membrane with embedded wire moves or the magnet moves.

## 3. MEMS Fabrication of Polymer-Based Actuator

### 3.1. Fabrication of EM Actuator

There are several mechanical actuation mechanisms related to the function of the membrane such as vibration, peristaltic and flexural plate wave [75]. Some actuators are constructed with flat movable membranes [45], some others are equipped with pillars or cilia, as found in micromixers [76]. For these purposes, certain MEMS fabrication methods with high resolution pattern are needed in order to create three-dimensional structures on the membrane. It should be noted that the fabricated membrane structure must be flexible enough to generate movement and able to withstand the pressure acting onto the surface. The patterned structures on the membrane were also predicted to improve the membrane’s flexibility.

The common method used in fabricating a polymer membrane with three-dimensional (3-D) structure is soft lithography or micro-molding. Soft lithography technique for polymer-based MEMS device was introduced in 1990 by Varadan [77]. Among the advantages of soft lithography techniques compared to conventional optical lithography techniques are the unlimited machining resolution of the emission and dispersion of optical waves and the turbulence in the resin. In addition, soft lithography with elastomer sealants has the advantage of precise pattern on the target surface and easy to remove from the mold. All of these advantages make soft lithography a great attractive and highly potential technique to be used in the field of microfabrication process [78].

Most of the polymer membranes fabricated through soft lithography technique do not have their own mechanism in order to function as an actuator. They need an external stimulation either from a permanent magnet or an electromagnet. Via this concept, an actuator disc, a magnetized permalloy strip, a bulk magnet or an embedded electromagnetic coil can be integrated into the polymer membrane structure to generate force for the membrane deformation purpose [79,80,81,82]. Soft lithography technique is not only an inexpensive and simple fabrication process but it can also manipulate the texture of the polymer membrane during fabrication to control its flexibility which is vital for membrane actuation [83,84,85].

**Figure 2:**
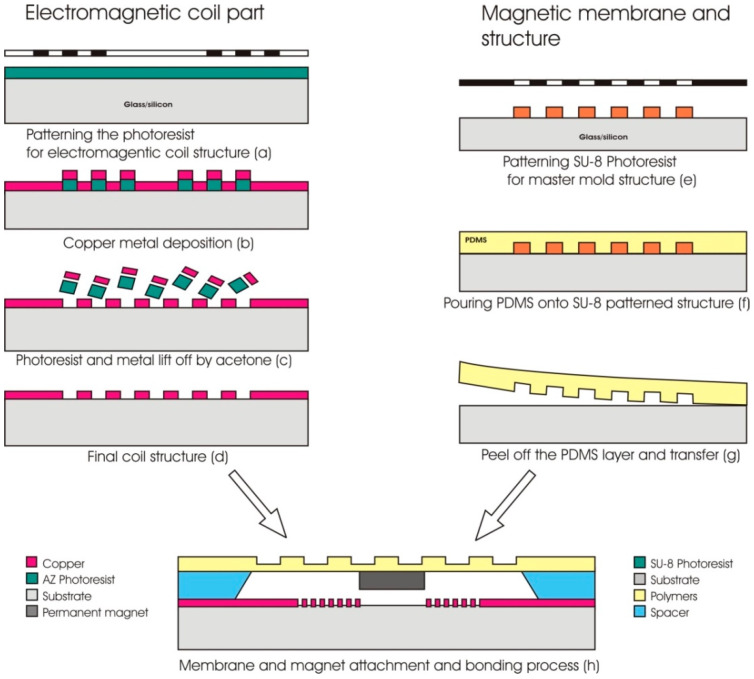
Schematic process step for the fabrication of polymer-based MEMS EM actuators with micro-pillar structures using the soft lithography process technique.

Some examples of soft lithography process in the fabrication of polymer-based MEMS structure were reported by Ghanbari et al. [86] and Yunas et al. [87,88]. The microactuator part can be fabricated separately. Thus, the fabrication process can start with the electromagnetic part (1), followed by the fabrication of magneto-mechanic part (2) and finally with the bonding of both parts using epoxy (3). The detailed fabrication process of a micropump system is shown in Figure 2. The electromagnetic coil pattern is first created followed by the deposition of planar copper (Cu) microcoil wire (a) and (b). The coil structure is formed after the lift-off process (c) and (d). Next is the fabrication of the magneto-mechanical part that involves the patterning of mold master using SU8-based photolithography process (e). Then, the polymer membrane is fabricated by pouring the PDMS onto the pre-patterned structure (f) followed by peeling-off of the material (g) before transferring it onto a spacer surface. The final step of the process is the attachment of the permanent magnet onto the transferred membrane and all fabricated parts are bonded together using epoxy glue (h).

Another approach to create membrane with 3-D (three dimensional) structures has been reported by Xu and Cui [89]. They used hot embossing technique to fabricate an actuator membrane by constructing the membrane layer-by-layer (LbL). In the process, silicon molds were fabricated using a conventional UV lithography and wet, etching technique. The hot press technique was then used to transfer the design structure from silicon molds to PMMA sheets. The hot press molding technique involves the simultaneous application of heat and pressure in the fabrication of a polymeric membrane.

Furthermore, 3-D structures can be created using 3-D printing technique that can print biocompatible polymers or devices at required dimensions based on the printer’s resolution. The technique offers more complex and sophisticated design that can be realized at micro-scale which could not be done with conventional method like soft lithography [90,91]. There have been also several studies reporting the usage of 3-D printing to fabricate a part of MEMS device such as the stereolithographic (SL) 3-D printer that fabricates a thin membrane from poly(ethylene diacrylate) resin [92]. The membrane was then pneumatically pressed to get the thickness smaller than 25 µm.

Zhou et al. [93] also reported in 2019, that a polymer actuator membrane with a thickness of 100 µm was successfully fabricated using a 3-D multijet printer (MJP). The printed membrane was able to deform in order to close and open the microchannel and fully functioning as a valve. Another novel 3D-printed electromagnetically driven fluidic valve was fabricated by projection–stereolithography (PSL) in combination with functional elements such as the permanent magnets [94]. There was also a study on the fabrication of a whole MEMS device using 3-D printing technique that met minimum the requirement for biocompatible standard [95].

### 3.2. Fabrication of Magnetic Polymer Composites Membrane

Embedded magnetic particles in polymer would be the future functional material for many types of biomedical devices. It becomes a new composite material that possesses the flexible mechanical characteristic and exceptional magnetic responsive features [96]. The implementation of magnetic polymer composite as the material structure for the actuator membrane could overcome the need of a bulk permanent magnet. The soft and flexible properties of polymeric membrane would tend to rupture and break when a bulk structure is placed on it, like the bulky permanent magnet attached onto an actuator membrane of a micropump [88].

One of the methods in fabricating magnetic polymer composite MEMS membrane is through a synthesis method using mechanical stirring under sonication in which a PDMS-based polymer was mixed with NdFeB magnetic particles having the size ranging from 50 to 100 um [9]. The deformation capability of the membrane has been tested, by which the highest deflection of 9.16 µm at 6 vol% magnetic particles density has been measured with an applied magnetic field density of only 0.98 mT.

Here, the PDMS is considered as the most popular material for flexible biomedical device applications. Apart from its biocompatible property, the mechanical properties can be manipulated via controlled ratio of the polymer base and curing agent [97]. The PDMS-based membrane has been successfully fabricated with the integration of the magnetic particles from 2% up to 30% distribution across the membrane. The magnetic membrane can be deflected when its magnetic field interacts with the magnetic flux formed from the current flow in the coils. The fluctuating movement of the membrane is governed by the applied current of only several milliamperes.

Recently, Tahmasebipour and Paknahad have fabricated nano-magnetic membrane made of PDMS–Fe_3_O_4_ for the application of valveless electromagnetic micropump [98]. Nano sized particles of Fe_3_O_4_ were mixed within the PDMS layer in order to create the magnetic membrane. The composite magnetic membrane is compatible with living tissues and has great magnetic stability. The embedding of nanoparticles in polymer however can cause agglomeration problem due to the attractive forces between the particles. Therefore, different approaches have been proposed to minimize particle agglomeration, such as particle encapsulation with polymeric material [99] or ceramic coating [100] or by implementation of surfactant [101].

## 4. Application of Polymers for Electromagnetic Actuators

### 4.1. Magnetic Polymer Composite-Based Microactuators

A flexible membrane with embedded magnetic particles having small particle size would have many advantages, because the magnetization and the magnetic anisotropy of the particles can be much greater than a bulk magnetic specimen [102]. The magnetic polymer composite is very light, hence would not significantly affect the mechanical properties of the polymer. Hence, this magnetic polymer composite membrane enables actuators to have larger deflection with a controllable actuation forces, compared to silicon or metal-based actuators [103]. On other hand, with the help of photo sensitive mold master material, the polymer composite would be able to be patterned and transferred onto the substrate as suspended movable part and other MEMS passive structures as well. Thus, the material composite can find its potential application as sensitive actuator for fluid injection, valves, magnetic recording media, mechanical relays, optical mirror and switch and other mechanically moving part driven by magnetic fields [15,104,105,106].

**Figure 3:**
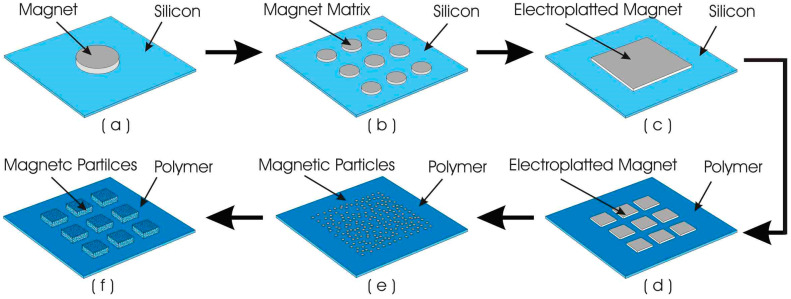
Development history of magnetic polymer composite-based MEMS actuator membranes, (**a**) silicon-based membrane with attached permanent magnet [71], (**b**) silicon membrane with attached small permanent magnet in matrix form [19], (**c**) silicon membrane with electroplated magnetic material [19], (**d**) polymer membrane with electroplated magnetic material [107,108], (**e**) polymer membrane with embedded magnetic particles [9], (**f**) polymer membrane with three-dimensional matrix structured embedded magnetic particles [88].

The evolution of magnetic material used for the actuator membrane shows a transition from bulk magnet to matrix magnet and now to magnetic polymer composite. The research of magnetism in electromagnetic actuator has then been extended by reducing the size of the magnetic particles embedded in the polymer membrane from micro to nanometer. Here, the evolution of the magnetic membrane is described in Figure 3.

Initially, silicon material was used as the membrane, which was bonded with a single bulky permanent magnet glued on the top of the membrane. Then, smaller permanent magnet with matrix structure was used to replace the single magnetic bulk in order to reduce the membrane stiffness. In 2002, the use of polymer material as the actuator membrane had been started and the permanent magnetic layer was created on the polymer membrane via electroplating [107]. The concept was then extended with the use of arrays of electroplated permanent magnetic layer [108]. Finally, the electroplated permanent magnet has then been replaced with the embedded magnetic particles, producing a magnetic polymer composite membrane with significant improved performances [88].

The current status of the magnetic polymer composite membrane for biomedical application was reported by Said et al. [88]. They developed a matrix patterned magnetic polymer composite for actuator membrane that is integrated with the micropump for bio-sample injection. The composite membrane is made of polydimethylsiloxane (PDMS) mixed with NdFeB magnetic particles and patterned into blocks of matrix.

**Figure 4:**
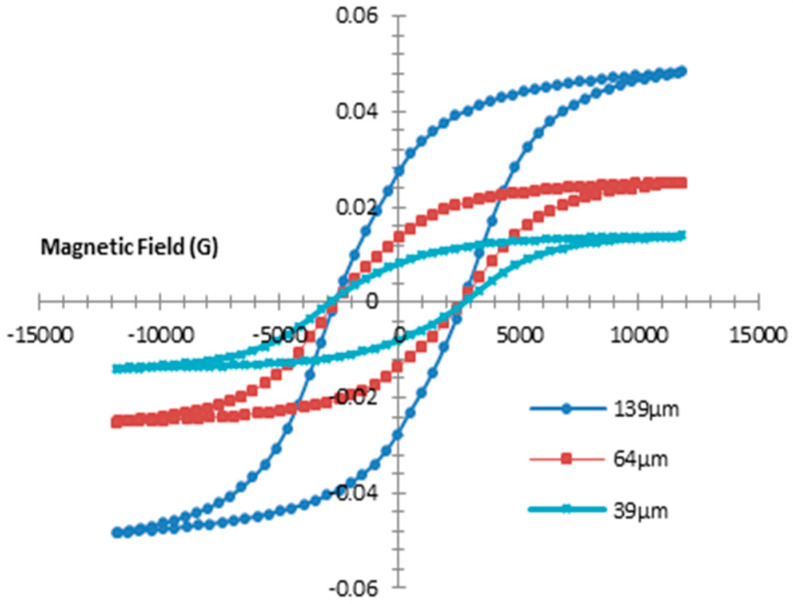
Hysteresis loop of 6% NdFeB polymer composite.

To this concept, the magnetic composite actuator membrane containing 6% NdFeB was capable of generating a maximum membrane deflection up to 12.87 µm [9]. As shown in Figure 4, the magnetic property of NdFeB polymer composite is strongly related to the amount of magnetic particles embedded in the polymer. Thicker polymer layer with more NdFeB particles produces larger magnetization. However it doesn’t affect the change in coercivity. A 139 µm membrane thickness shows a saturated remanence magnet of 37.637 mT.

Some other potential applications of magnetic polymer composite in sensors and actuators were reported by Samaniego et al. [109]. They studied the resultant of magnetic polymer composite to fabricate soft robots by squeegee–coating method. The soft robots have flexible and compliant bodies resulting in higher degrees of freedom and improved adaptability to their surroundings. Therefore, the robot can be used for minimal invasive surgery (MIS) in order to reduce patients’ trauma, pain and recovery time [110]. The soft polymer-based magnetic actuator was fabricated by mixing ferromagnetic microparticles (PrFeB) with polymers precursor before its curing. The soft robots were magnetized under 1 T of uniform magnetic field.

The magnetic polymer composite can also be integrated to the artificial cilia in a microfluidic system. Zhang et al. demonstrated the versatile microfluidic flow generated by molded Magnetic Artificial Cilia (MAC) [111]. The MAC can cause versatile flows by changing the magnetic actuation mode. This on chip microfluidic transport does not require tubing or electrical connections, reducing the consumption of reagents by minimizing the “dead volumes”, avoiding undesirable electrical effects and accommodating a wide range of different fluids.

### 4.2. Polymer-Based Electromagnetic Actuators for Micropumps and Microvalves

Most important properties of polymer in micropump system as the movable membrane of the actuator are its flexibility and high surface strength. In general, microfluidic systems are made up of input and output tubes, channels and pump chambers. Beside the membrane, the valves are also important in ensuring fluid flow direction and to regulate the flow rate. Some other micropumps are designed valveless that improve the reliability of the system and reduces the clogging effect [112].

Wang et al. [113] reported a micropump comprising a magnetic PDMS diaphragm, a planar microcoil and microfluidic channel. When an electric current is applied to the microcoil, an electromagnetic force is generated at the magnetic diaphragm. The deflection of PDMS diaphragm generate a push–pull action of the membrane hence creating pressure difference within the chamber and microchannel and subsequently causing the fluid flow. Their EM micropump achieved a maximum pumping rate of 52.8 µL/min with diaphragm displacement of 31.5 µm induced by a microcoil input current of 0.5 A.

EM micropump using PDMS encapsulation layer mounted with small permanent magnets was reported by Pan et al. [114]. The membrane of the pump actuator which is driven by magnetic motor shaft or microcoil used two one-way check valve using a micropipette and heat sink tubing. The magnetic motor shaft was a small DC motor (6 mm in diameter and 15 mm in length) with a neodymium–iron–boron permanent magnet embedded in its shaft. The EM micropump achieved a maximum pumping rate of 774 µL/min with magnetic motor shaft and 1 mL/min with microcoil driven pump. Microcoil driven pump has shown higher flowrate and much higher power consumption.

**Figure 5:**
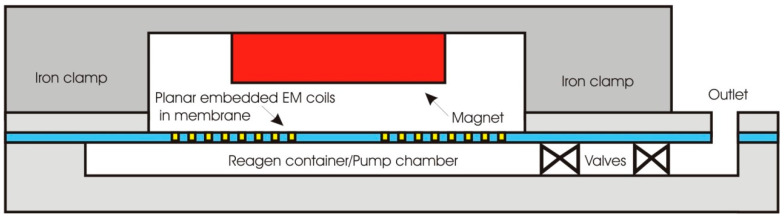
Polydimethylsiloxane (PDMS)-based EM micropump and valves with embedded planar microcoil.

Furthermore, an EM micropump with embedded planar coil in the thin PDMS membrane reported by Yin et al. (Figure 5) [115]. The size of the membrane is 7 mm in diameter and achieved 50 µm deflection with an applied current of 500 mA. The resonant frequency is about 1.43 kHz. Fluids in the microfluidic chip were driven forward by a local pneumatic pressure provided by the membrane. This EM pump was claimed to have a pumping volume flow rate of 2 µL/min.

**Figure 6:**
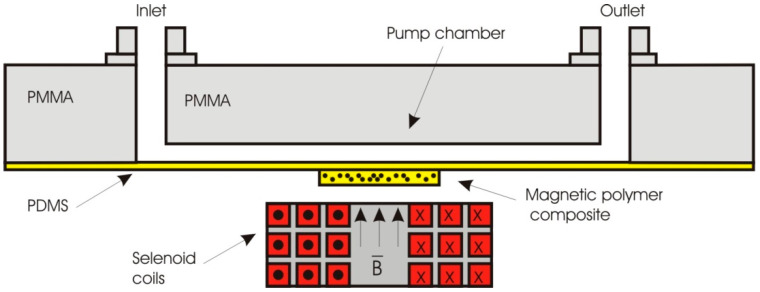
Schematic of a valveless EM micropump involving PDMS and PMMA materials and utilizing magnetic composite membrane to replace the bulk permanent magnet.

A valveless EM micropump reported by Yamahata et al. [60] used composite magnets replacing the bulk permanent magnets (Figure 6). The composites magnet was developed using PDMS polymer mixed with 40% of NdFeB powder with a mean size of 200 µm. The actuator was driven by a 1500 turns coil supplied with sinusoidal current of 150 mA with soft magnetic core in the center to strengthen the magnetic field. The actuator membrane could deform up to 0.25 mm and pumping water at the flowrate of 400 µL/min with resonant frequency of 12 Hz by applying nozzle/diffuser microfluidic system. The passive structure of the pump system is made of four PMMA layers consisting of capping layer, channel and chamber layer and also spacing layer.

Another valveless EM micropump having a composite magnetic membrane of Fe + PDMS was reported by Nagel et al. [116]. Weight concentration varying from 25%–75% of iron particles with the size of below 10 µm were mixed in PDMS. Nickel coated NdFeB magnet was used to interact with the actuator membrane and moved up/down by a crankshaft. This micropump used valveless microfluidic system with a 6-mm diffuser/nozzle that has produced a maximum pumping flowrate of 35 µL/min at a frequency of 1.67 Hz.

**Figure 7:**
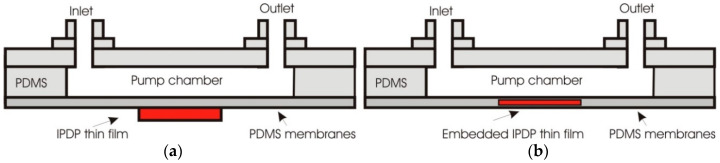
Schematic of iron particle dispersed PDMS (IPDP) for valveless micropumps, (**a**) stacked thin film design, (**b**) embedded thin film design.

Shen and Liu fabricated a PDMS-based magnetic composite membrane with IPDP thin film stacked design (Figure 7a) and embedded system (Figure 7b) [117]). An iron-particle-dispersed PDMS (IPDP) was a mixture of iron particles with the average size of 55 µm, PDMS and its curing agent. The mixing ratio of IPDP was 10:10:1. The micropump used a valveless type microfluidic system and electromagnet block which connected to power supply and combiflex. Micropump with IPDP embedded design had a pumping flowrate of 1.623 mL/min at a frequency of 6–7 Hz and 30 V of supply voltage which was higher compared to the flowrate of stack design.

A complete PDMS-based micropump including the structure, the membrane and the valveless microfluidic system was reported by Zhou and Amirouche [63]. The actuator membrane used a thin NdFeB magnet encapsulated at the center of the PDMS. Maximum deflection of the actuator membrane was 36.36 µm. DI water is pumped with maximum flow rate of 319.6 µL/min at a frequency of 36.9 Hz with supply current of 0.18 A.

Said et al. [118] combined the bulky permanent magnet with magnetic composite membrane to improve the reliability of the membrane and at the same time to increase the magnetic induction. The hybrid structure could produce a magnetic flux density of 37.637 mT enabling a controllable peristaltic pumping of microfluidic sample with a flowrate of 6.6 µL/min. When the bulk permanent magnet was removed from the micropump system and only left with the flat membrane composite alone, the micropump produced a very slow flowrate of 6.52 nL/min. Hence, the micropump could deliver a very precise dose for drug delivery system.

**Figure 8:**
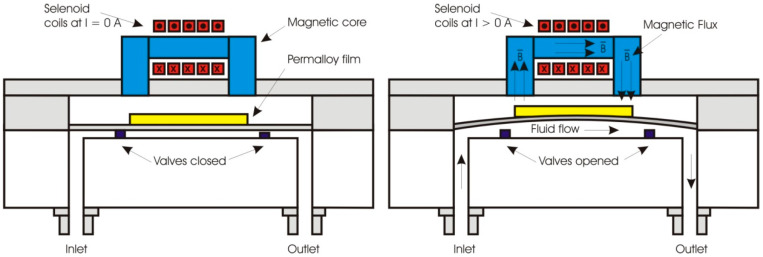
Electromagnetic actuator incorporating magnetic valves, (**left**) closed mode, (**right**) open mode.

Electromagnetically actuation of flexible membrane incorporating microvalve for micropump application has been also reported by Sadler et al., as shown in Figure 8 (left) in a closed mode and (right) in an open mode [119]. The normally closed magnetic microvalve has both fluidic and electrical connections bonded to a glass motherboard. The microvalve comprised a magnetoactive membrane, a stationary valve seat and inlet/outlet channel. The magnetoactive membrane as a diaphragm plated with permalloy films on the top will interact with electromagnet flux generated by inductor to produce the magnetic force. A polymer film was attached to the system to ensure there is no leak. The force would lift up the membrane from the valve seat thus opened the valve and allowed the fluids to flow from inlet to outlet due to pressure difference.

Polymer-based microvalves used to manipulate the fluid flow have been reported by Gaspar et al. [120]. The actuation of the valve is based on the principle that flexible polymer walls of a liquid channel can be pressed together by the aid of a permanent magnet and a small metal bar. In the presence of a small NdFeB magnet lying below the channel of interest, the metal bar is pulled downward simultaneously pushing the thin layer of PDMS down thereby closing the channel and stopping any flow of fluid. Furthermore, Bute et al. [121] reported that the flow manipulation and proper operation of the valve depends on thickness and percentage load of magnetic material in the membrane as well as dimensions of channel, chamber and membrane with respect to the location of outlet channels, while Nakahara et al. [122] reported the use of photosensitive polymer composites for the fabrication of magnetically driven microvalve arrays in a µTAS (µ- total analysis system)

To summarize, since 1995 there have been many developments in microactuator device design and materials for micropumps and microvalves incorporating elastic membrane, as listed in Table 4. The magnetic polymer-based micropumps are working with various applied frequency and various reading fluid flow rates ranging from 6.5 nL/min to 6.8 mL/min have been obtained. Obviously it is found that after the use of silicon, polymers have become the subject of researcher’s choice to build the actuator membrane for micropump. Since 2005, magnetic polymers composites have become the promising material to replace the conventional bulky permanent magnet.

It can be concluded that the polymer-based micropump and valves were able to precisely control the delivery of the fluidic sample and obtained fluid flow range from 10 mL/min down to several nL/min. Innovations in the membrane material and structure and the use of the latest technology in several ways are still necessary to meet the needs and requirements of the biomedical instrumentations.

### 4.3. Polymer-Based Active Micromixer

Magnetic polymers found its important role in microfluidic mixer system which is mostly used as the basic material for the passive part of the system such as the channel and chamber formation in lab on chip system. On the other hand, the polymer has been playing the potential role as an active part in order to improve the mixing performance of the microfluidic system, especially for the bio-cell analysis. This is called active microfluidic mixer, which means that the mixing mechanism is due to the turbulences of the fluidic sample inside the mixer chamber, usually based on magneto-hydrodynamic and magnetic structure actuation, which is driven by an external magnetic field [128].

In 2018, Tang et al. presented a research on embedded flexible magnets in PDMS membrane [129]. Three designs were introduced and compared, namely (a) concentric type with the magnetic material in the center of the membrane; (b) eccentric type with the magnetic material offset from the center of the membrane and (c) split type with two regions of magnetic materials with opposing polarities. Oscillating fluid flow was induced at a frequency of 100 Hz to enhance mixing performance. Split type design proved to have better mixing performance than the others.

Turbulence inside the fluidic chamber to improve mixing performance can also be produced by using pillars rotation as reported by Rahbar et al. [76]. Here, an individual or arrays of micromixer element in form of high aspect ratio of small pillar was fabricated using a micromolding process of nanomagnetic-composite polymers. The cilia, which are realized in PDMS (polydimethylsiloxane) doped with (Nd_0.7_Ce_0.3_)_10.5_Fe_83.9_B_5.6_ powder are then magnetized to produce permanent magnetic structures with bidirectional deflection capabilities, making them highly suitable as mixers controlled by electromagnetic fields. Similar to this concept, Zhou et al. [130] reported the development of magnetic pillars made of polymers composite with embedded Fe_3_O_4_ magnetic particles. Through an external magnetic field exposure, the magnetic pillar will react in rotation mode.

**Figure 9:**
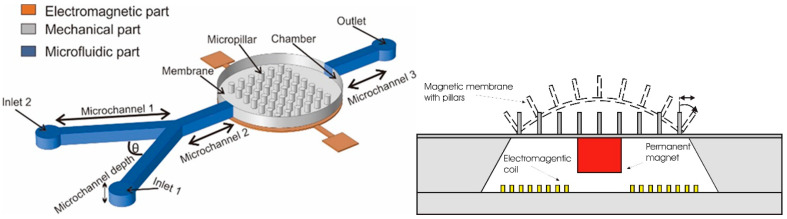
Schematic diagram of a pillar-based active microfluidic mixer device (**left**) and the micromixer structure showing the polymer pillars swing profile following the deformation of the actuator membrane (**right**).

Furthermore, Pawinanto et al., [131] has developed polymer pillars on a movable flexible magnetic membrane with an attached permanent magnet (Figure 9, left). The movement of the pillars followed the deformation profile of the membrane (Figure 9, right). The concept of pillar rotations or membrane with pillar deformation in a mixer chamber has significantly increased the index of turbulence enabling higher mixing efficiency. These improvements thank to the advancement in the fabrication method of active micromixer that simplifies the mixer structure and its fabrication.

## 5. Conclusions and Future Aspects of Polymers for EM Actuator

In this study, a comprehensive review on electromagnetically driven MEMS actuators with polymer-based movable structure is presented. The flexible characteristic of polymer is beneficial in attaining large and controlled structure deformation of the movable parts, such as thin membrane diaphragms, pillars or cantilevers. Significant discovery of polymer-based functional material has led to a wide range application of electromagnetic MEMS actuator. The flexible actuator structure with high magnetic property plays an important role in various biomedical instrumentations, such as lab on chip and drug delivery system.

These actuators can function as micropumps, microvalves and micromixers which execute the imperative roles of delivering biomedical samples. The wonderful combination between flexibility and magnetic properties of the magnetic polymer actuators can accurately control the microfluidic flow in a microchannel and determine its direction. In addition, the fluidic samples can be delivered precisely at a wide range of fluid flow rate, from 30 mL/min down to several nL/min. It will be a challenging effort to widen the flow rate range of an electromagnetic injection system which may require significant arrangement in pump and valve system design. This will eventually improve the reliability and quality of the electromagnetically driven microactuator system, specifically designed for drug delivery and artificial kidney.

The actuator structure also plays an important role as an active microfluidic mixer in the preparation process of the biomedical samples for drug delivery and lab on chip system. The polymer actuator can potentially reduce the mixing time and increase the mixing index. The increase of fluid sample turbulence inside the mixer chamber driven by external magnetic fields improves the mixing performance. Here, the innovation in design and fabrication technology for magnetic polymers introduces more compact mixer structure.

The presence of bulk permanent magnet attached onto the actuator has been identified as one of the main drawbacks for making an electromagnetically driven MEMS actuator to be large in size. Hence, it is crucial to make the actuator structure compact, as this will ultimately reduce the overall size of the system. A polymer membrane diaphragm with embedded magnetic nanoparticles can become a compact actuator with better mechanical properties. The developmental concept for magnetic actuator has evolved from the utilization of hard and fragile materials to more flexible polymeric materials with matrix magnet and now progresses towards embedded magnetic nanoparticles polymer composites. In the future, the polymer composites will eliminate the need of a conventional bulky permanent magnet in electromagnetic actuators.

To conclude, much progress has been made on magnetic actuator development and the future trend shows magnetic polymer composites as the new functional materials for flexible biomedical device technology. The magnetic polymer composite will be a fascinating material to be implemented in wearable and portable biomedical device applications that are currently and rapidly growing.

## Figures and Tables

**Table 1:** Material properties of popular polymers used in microelectromechanical systems (MEMS).

Polymer Name	Density	Young’s Modulus (GPa)	Poisson’s Ratio	Thermal Expansion Coefficient @25 °C (10^−6^ K^−1^)	Thermal Conductivity (W/mK)	Property Utilized	Process
PMMA [41,42]	1.17–1.2	3.1–3.3	0.35	70–90	0.186	Little elasticity, optical property	LIGA, Hot embossing
Parylene [43]	1.289	4.5	0.4	35	-	Vapor barrier	Coating
PDMS [29,39,44]	0.97	0.36–0.87	0.5	310	0.18	Elasticity	Molding
Polyimide [45,46,47]	1.42	3	0.34	30–60	0.1–0.35	Little elasticity	Coating

**Table 2:** Typical MEMS mechanical actuator devices, structure and their working principle.

Working Principle	Schematic of Actuator System	Advantages	Disadvantages	Typical Applications
Piezoelectric [9,50,51]	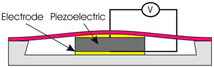	High pressure Fast response	Complicated process High input voltage, low reliability	Micropump, microvalve, microgripper
Electrostatic [52,53,54,55]	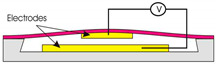	Low Power Fast response Controlled large deformation through input voltage	Small membrane deformation, low reliability	Micromotor, microshutter, micromirror microrelay, micropump
**Electromagnetic [9,12,49,56]**	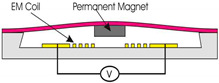	**High pressure.** **High membrane deformation** **Easy control thrugh input current** **Fast response** **Large frequency range.**	**Large size.** **Thermal effect**	**Micromotors, micro relay, switch, micro pump, valve, mixers, microspeaker and magnetostrictive**
Polymer composite Electroactive [57]	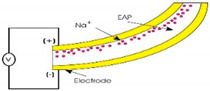	High deformation Low power Ability to work at wet environment Low footprint	New actuation mechanism. Complicated structure and process Very limited application	Micro robotic, micromanipulators
Thermo-pneumatic [11,58]	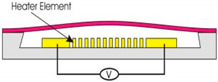	High pressure	Specific material high power consumption long response time. Limited application	Micropump, microvalve, inkjet printhead

**Table 3:** Common magnetic actuator devices used for biomedical applications.

References	Actuating Element (Structure, Material or Method)	Magnet Type	Input	Specifications
**Biosample Delivery and Transport**				
Yamahata et al. 2015 [60]	PDMS membrane & magnet	Iron powder	33–150 mA	Flowrate: 0.4–1.6 mL/min Frequency: 6–12 Hz
Büttgenbach, 2014 [61]	EM Micromotor rotation & polymer magnet	90 wt% ceramic ferrites + polymer	70 mA	Forces: 1.2 mN Torque: 10 μNm
Lee et al. 2011 [62]	Silicon catheter	Electroplated nickel	70 to 1500 Hz (resonant frequency)	Angle > 60°
Zhou & Amirouche, 2011 [63]	PDMS membrane & magnet	NdFeB or CoNiMnP plate	90–180 mA	Magnetic Force: 16 µN Flowrate: 319.6 µL Frequency: 36.9 Hz
**Biosample Preparation**				
Nouri et al. 2017 [64]	Magnetohydrodynamic interaction with permanent magnet	Fe_3_O_4_ nanoparticles	3000 Gauss	Mixing time: 80 s Mixing index: 0.9 s
Liu et al. 2016 [65]	PDMS with permanent magnet	Magnetic composite (carbonyl iron)	6 V, 18 Hz	Mixing time: 2 min Flow rate: 20 μL/s
**Biocell and Drug Particles Manipulation**				
Banis et al. 2020 [66]	water-soluble ferrofluid material (FluidMAG lipid)	Electromagnetic coils	4 to 8 A Magnetic particle size 100 nm	Droplet velocity 135 µm/s
Rinklin et al. 2016 [67]	Magnetophoretic attraction of microbeads	carboxyl functionalized particles (Dynabeads) and laminated magnetic NiFe parts	5, 10 and 15 mA	Maximum particle levitation height of approximately 10 μm
Chen et al. 2015 [68]	PDMS tweezer with hexapole yoke	10 layers of laminated magnetic NiFe parts	feedback control at a speed of up to 1 kHz	Maximum force = 400 pN, force distribution with actuation from −30 µm to 30 µm
Choi et al. 2000 [69]	silicon cantilever	Encapsulated permalloy	N/A	N/A

**Table 4:** Development of polymer-based MEMS electromagnetic actuators for microfluidic pump applications.

Year	Membrane Structure	Flowrate	Frequency	References
1995	Thermoplastic molding bulk permanent magnet	780 µL/min	5 Hz	Dario et al. [123]
1999	Silicon rubber	2.1 mL/min	50 Hz	Bohm et al. [124]
2000	PDMS + plate alloy	1.2 µL/min	2.9 Hz	Khoo dan Liu [125]
2005	PDMS + bulk permanent magnet	774 µL/min	n.a	T.Pan et al. [114]
2005	PMMA and composite PDMS + powder NdFeB	400 µL/min	12 Hz	Yamahata et al. [60]
2006	Composite PDMS + powder Fe	35 µL/min	1.73 Hz	Nagel [116]
2007	PDMS + bulk permanent magnet	2 µL/min	n.a	Yin et al. [115]
2008	PDMS + bulk magnet NdFeB and PMMA	6.8 mL/min	20 Hz	M.Shen et al. [126]
2010	Composite PDMS + powder Fe	1.623 mL/min	6–7 Hz	Shen and Liu [117]
2011	Composite PDMS + plated NdFeB	319.6 µL/min	36.9 Hz	Zhou and Amirouche [63]
2015	PDMS + magnet pad	n.a	28–30 Hz	Dich et al. [127]
2017	Composite PDMS + NdFeB particles	6.52 nL/min	1 Hz	Said et al. [88,118]
